# The Successful Management of Primary Amenorrhea in Woodhouse–Sakati Syndrome: A Case Report and a Literature Review

**DOI:** 10.3390/life13102022

**Published:** 2023-10-07

**Authors:** Hanadi Bakhsh, Norah Alqntash, Ebtesam Almajed

**Affiliations:** 1 Clinical Sciences Department, College of Medicine, Princess Nourah Bint Abdulrahman University, Riyadh 11564, Saudi Arabia8ebtesamalmajed@gmail.com (E.A.); 2 Department of Obstetrics and Gynecology, King Abdullah bin Abdulaziz University Hospital, Princess Nourah Bint Abdulrahman University, Riyadh 11564, Saudi Arabia

**Keywords:** Woodhouse–Sakati syndrome (WSS), primary amenorrhea, alopecia, hormonal replacement therapy (HRT)

## Abstract

Background: Woodhouse–Sakati syndrome (WSS) is a rare multisystemic disease resulting from an autosomal recessive gene mutation characterized by distinctive facial appearance, alopecia, impaired HbA1c, and hypogonadism. Purpose: To present the successful management of primary amenorrhea in a WSS patient. Case Presentation: We report a 19-year-old Saudi female referred to the gynecology clinic at the age of 16 as a case of primary amenorrhea. The patient underwent a genetic analysis, which revealed mutations in the DCAF17 gene, confirming the diagnosis of WSS. Treatment includes hormonal replacement therapy for the induction of puberty. Conclusions: Careful and detailed medical and physical examination led to appropriate testing confirming the WSS diagnosis. Genetic tests for family members and the offspring of the patient are strongly recommended. Treatment timing and dosage are determined by the patient’s individual needs, which take into consideration the patient’s potential for growth, the family’s readiness, and any comorbidities.

## 1. Introduction

Woodhouse–Sakati syndrome (WSS) is a rare multisystemic disease resulting from an autosomal recessive gene mutation, described for the first time by Woodhouse and Sakati in 1983 following the observation of six Saudi Arabian patients from two highly inbred families [1,2]. From 1983 to the present, the number of patients with WSS has increased to 76 from 32 families, representing various geographical areas but mainly from the Middle East. Several other cases have been reported in Europe, Turkey, Japan, Portugal, Pakistan, and India [3].

The characteristic features of WSS include a distinctive facial appearance (i.e., prominent nasal bridge, hypertelorism, and a long triangular face), sparse or absent hair, hypogonadism, diabetes mellitus, intellectual disability, mild sensorineural deafness, progressive extrapyramidal movements (i.e., dystonic spasms with dystonic posturing, dysarthria, and dysphagia), variable changes in S-T and T wave abnormalities on an electrocardiogram, and a decreased insulin-like growth factor 1 level [1,4]. Alopecia and hypogonadism are the hallmarks of the syndrome [5]. Mutations in the *DCAF17* (previously known as *c2orf37*) gene were identified as the cause of the molecular pathology of WSS [6].

WSS is characterized by a wide range of clinical features, and the severity and combination of these features can vary significantly from one individual to another. This variability means that the prognosis can differ widely among affected individuals. Some may experience milder symptoms that are manageable, while others may have more severe and debilitating manifestations [7]. Several aspects of WSS, such as the extrapyramidal movements, diabetes mellitus, and intellectual disability, tend to be progressive. This means that symptoms may worsen over time, potentially leading to increased disability and healthcare needs [8]. Progressive symptoms can have a significant impact on the long-term prognosis [9].

One of the hallmark features of WSS is hypogonadism, which refers to underdeveloped or nonfunctional reproductive organs [10]. In females with WSS, hypogonadism can manifest as delayed or absent puberty, which is a leading cause of primary amenorrhea. The genetic mutations responsible for WSS can affect the normal development and functioning of the ovaries and other reproductive structures [11].

WSS can lead to hormonal imbalances, including disruptions in the hypothalamic-pituitary-gonadal axis, which plays a crucial role in regulating the menstrual cycle. These hormonal disturbances can result in the absence of menstruation, contributing to primary amenorrhea. Many individuals with WSS experience delayed puberty due to the effects of hypogonadism and hormonal imbalances. Delayed puberty often includes a delay in the onset of menstruation, leading to primary amenorrhea [12].

The management of primary amenorrhea in individuals with WSS typically involves a multidisciplinary approach. Endocrinologists, gynecologists, and geneticists may collaborate to diagnose the underlying cause and develop a tailored treatment plan. Hormone replacement therapy (HRT) is often considered to induce puberty and menstruation in affected females [13]. Genetic counseling is essential for families affected by WSS. Understanding the genetic basis of the syndrome can help individuals and their families make informed decisions regarding family planning and provide insights into the likelihood of passing on the syndrome to future generations [14].

While there are limited data on life expectancy for individuals with WSS, it is generally believed that individuals with this syndrome have a normal lifespan. However, the presence of diabetes mellitus and associated complications, as well as the potential for movement disorders and their impact on daily functioning can influence the overall quality of life and life expectancy [15]. The prognosis for quality of life in individuals with WSS is highly dependent on the management of the syndrome’s various clinical manifestations. Early and comprehensive medical and therapeutic interventions can improve the quality of life for affected individuals [16].

The purpose of this study is to describe the successful management of primary amenorrhea in a patient with Woodhouse–Sakati syndrome. This study will describe the clinical, laboratory, genetic, and radiological investigations performed following a patient’s referral to our clinic and her management plan over the past four years.

## 2. Case Report

The patient is a 19-year-old Saudi female referred to the gynecology clinic at age 16 as a case of primary amenorrhea. Aside from some intellectual difficulties noticed when she enrolled in the school, she had a relatively uneventful childhood. She is a known case of myopia of more than −3, diagnosed at the age of eleven without evidence of retinopathy. She was born by spontaneous vaginal delivery at term without any perinatal or postnatal complications. The patient’s parents are first-degree relatives and have five daughters, all of whom are healthy and medically free, except for our patient. Her mother and sister had menarche at ages 12 and 13, respectively. The patient did not complain of hearing loss, dysarthria, dysphagia, or lower limb dystonia at any point in her life. She met all her developmental milestones as expected.

During the general examination, the patient was alert and oriented, and vital signs were stable. She was obese, with a height of 154 cm and a weight of 83.7 kg (BMI 35 kg/m^2^). On focus examination, the patient was found to have sparse, thin hair, thin eyebrows, and proptosis. In addition, she exhibited underdeveloped secondary sexual characteristics, Tanner stage B1 P1. Furthermore, a soft systolic murmur was found in the left upper sternal border. Dysmorphic facial features, including a triangular face, prominent nasal bridge, and hypertelorism, were present. The examination of the rest of the other systems was unremarkable. The clinical features of our case are presented in Table 1 along with clinically diagnosed cases reported in the literature.

On presentation, the laboratory findings of routine investigations (i.e., complete blood count (CBC) with differential) were normal, with slightly elevated glycated hemoglobin levels (HbA1c) (6.8% (reference range 4.4–6.4%)). The thyroid profile of the patient revealed normal thyroid stimulating hormone (TSH) (3.266 uIU/mL (reference range 0.38–5.33 uIU/mL)) and normal free thyroxine (FT4) level (13.50 pmol/L (reference range 7.50–21.10 pmol/L)). The patient’s hormonal profile demonstrated a high follicle-stimulating hormone (48.5 mIU/L, normal range 3.85–8.78 mIU/L) and high luteinizing hormone (17.96 mIU/L, normal range 2.12–10.89 mIU/L). These laboratory results indicate a combination of hypergonadotropic hypogonadism and impaired HbA1c.

The patients underwent further diagnostic genetic testing. Based on the results of karyotyping, 46, XX was determined. The results of the chromosomal microarray analysis (CMA) were negative. An NGS analysis revealed a homozygous pathogenic variant in the DCAF17 gene. A genetic diagnosis of autosomal recessive WSS has been confirmed. Table 2 summarizes the important laboratory findings between the ages of 15 and 19.

**Figure 1:**
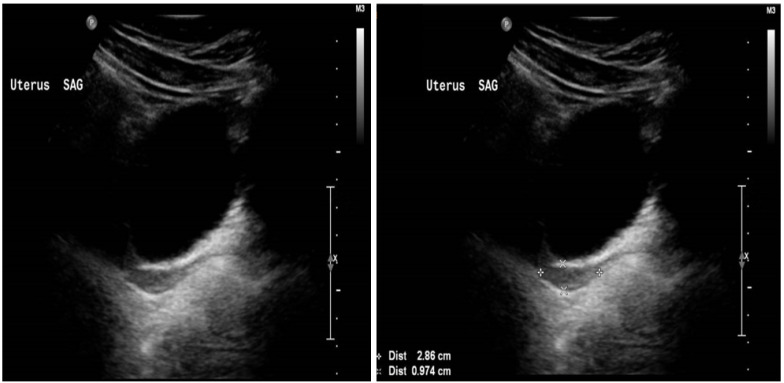
Ultrasound of the uterus at presentation when the patient aged 15 years.

At presentation, the patient’s pelvic ultrasound demonstrated the uterus to be very small, measuring about 3.0 × 1.7 × 0.5 cm homogenous echo pattern and smooth outline, with no focal lesion. The endometrium was identified, and small ovaries with no definite follicles were seen. The right ovary measured 1.1 cm × 1.2 cm × 0.5 cm (volume 0.40 mL). The left ovary measured 1.3 cm × 1.1 cm × 0.7 cm (volume 0.53 mL), as demonstrated in Figure 1.

The management plan for primary amenorrhea and the absence of secondary sexual characteristics (hypergonadotropic hypogonadism) included three phases: phase (1): induction of pubertal breast development; phase (2): establishment of normal regular menses and acquisition of normal bone mineralization; and phase (3): long-term maintenance of a normal estrogen state.

**Figure 2:**
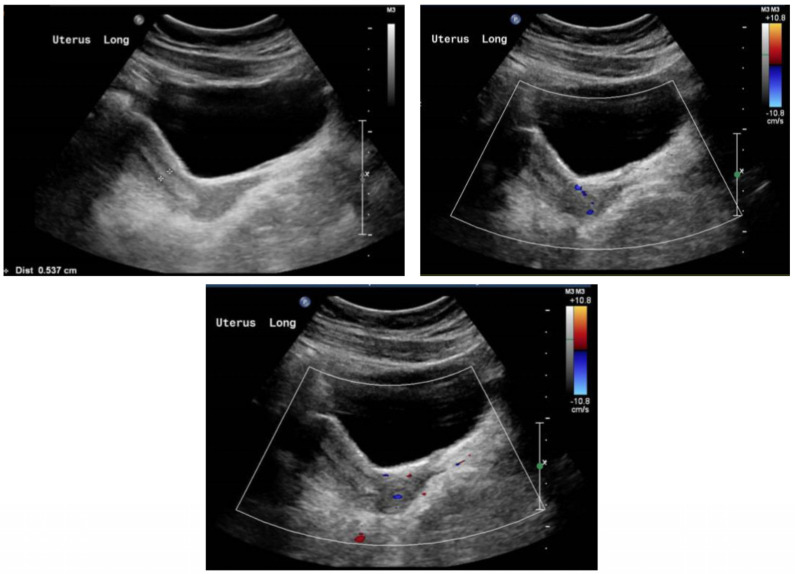
Ultrasound of the uterus following the management plan at 19 years old.

During the first phase of treatment, estrogen monotherapy (Premarin^®^ (conjugated estrogen) 0.3 mg PO OD) was prescribed, starting at a low dose of 0.3 mg daily, with regular follow-ups by pelvic ultrasounds to determine the uterine size and physical examinations to determine the Tanner stages of breast development. The dose of estrogen monotherapy was gradually increased over time based on the uterine size and breast development. Six months following the completion of phase 1 therapy, the dose of estrogen monotherapy was increased to enhance breast development and induce menses. After 18 months of estrogen monotherapy, oral progestin therapy (dydrogesterone 10 mg) was added during phase 2 to maintain endometrial health. Dydrogesterone was given daily for the first five days of the month. Thereafter, the patient experienced menstruation. The pelvis ultrasound of the patient at the age of 19 demonstrated an anteverted uterus of normal size, measuring about 6.2 cm × 2.3 cm × 3.1 cm, homogenous echo pattern, and smooth outline. The endometrial thickness was about 0.6 cm. The right ovary measured about 1.8 cm × 1.0 cm × 1.4 cm (volume 1.3 mL). The left ovary measured 2.4 cm × 2.2 cm ×1.5 cm (volume 4.0 mL), as shown in Figure 2. Both ovaries were relatively small, showing multiple small follicles with no dominant follicle. There was obvious solid or cystic mass lesion.

Within the first six months, the Tanner stage of breast development evolved into the B4. Then, the dydrogesterone course increased to 10 days each month. Long-term maintenance doses of estrogen were provided, plus progestin for 12 to 14 days each month to protect the endometrium from endometrial hyperplasia.

## 3. Discussion

We reported a homozygous pathogenic variant in the *DCAF17* gene, leading to Woodhouse–Sakti syndrome, in a 19-year-old female with consanguineous Saudi parents. The most salient features, in this case, were intellectual disability, alopecia, absence of secondary sexual characteristics, and primary amenorrhea. Unlike most cases, our patient did not present with diabetes. Moreover, she did not present with sensorineural hearing loss, extrapyramidal symptoms, or hypothyroidism.

Based on a literate review article of 58 Qatari cases with WSS, it was noted that all had alopecia and hypogonadism, and only 46% had diabetes [3]. Comparatively, according to a systematic review by Agopiantz et al., it was found that all 72 patients with WSS manifested alopecia, hypogonadism, and decreased insulin growth factor-1 (IGF-1) levels, with the latter possibly being associated with the development of diabetes mellitus [7]. To be precise, diabetes and extrapyramidal symptoms usually appear later in life, in early adulthood, rather than earlier on in childhood [20]. This could explain the absence of these features in our case.

As the patient was born to consanguineous parents who were both in good health, this indicates that the syndrome is inherited by autosomal recessive means. Moreover, the patient had an uneventful childhood with a normal development and growth path. These findings were in line with a previous report on two sisters with the same syndrome [21]. Moreover, we reported a mutation in the *DCAF17* gene. This mutation was first mapped by Alazami et al. [22], where they identified a biallelic founder variant in the gene C2orf37 (now known as DCAF17) encoding a nucleolar protein on chromosome 2q22.3–2q35 in several Saudi consanguineous families showing characteristic features of WSS. This gene is expressed in the skin, brain, and liver, which correlates with organ involvement in patients with WSS.

Primary amenorrhea occurs when menarche fails to occur by the age of 16 in the presence of normal growth and secondary sexual characteristics. When menstruation has not occurred by the age of 13, and puberty-related characteristics, such as breast development, have not manifested, a workup for primary amenorrhea should be conducted. It is estimated that 48.5% of primary amenorrhea cases are caused by hypergonadotropic hypogonadism (POI), 27.8% by hypogonadotropic hypogonadism, and 23.7% by eugonadism (pubertal delay with normal gonadotropins; 23.7%) [23].

The exact pathophysiology is unclear, but it likely involves hypothalamic–pituitary–gonadal axis dysfunction leading to a lack of menstrual cycles. Hypergonadotropic hypogonadism causing ovarian failure is a predominant mechanism. Amenorrhea manifesting as delayed or absent puberty requires detailed evaluation and treatment to induce the development of secondary sexual characteristics, cyclic menstruation, and prevent future complications like osteoporosis [24].

The diagnosis of WSS requires a high index of clinical suspicion based on phenotypic features and detailed history. Alopecia and hypogonadism are the most consistent findings [25]. While exome sequencing can confirm DCAF17 mutations, targeted gene panels for intellectual disability or amenorrhea may also assist the diagnosis. Prenatal testing and genetic counseling enables recurrence risk assessment. Baseline evaluation should include IQ assessment, endocrine workup, hearing and ophthalmic examination. Regular developmental monitoring, lifestyle modification and family support are integral [26].

Treatment is tailored to symptom control, given the lack of curative therapies. Hormone replacement regimens need to be individualized based on growth potential and comorbidities. Diabetes management with lifestyle measures, insulin sensitizers and other oral hypoglycemic agents is warranted. Monitoring for emergence of movement disorders, thyroid dysfunction and psychiatric manifestations is key [27]. Physical therapy can help with motor impairment. Psychosocial support and skill development should be offered. A multidisciplinary approach is ideal to optimize quality of life. Future studies on genotype-phenotype correlation, natural history and standardized management guidelines are required to improve prognosis. The treatment of WSS patients is currently individualized and managed in a multi-disciplinary pattern [28].

This case report provides valuable insights into the management of primary amenorrhea in the rare Woodhouse–Sakati syndrome, for which there is limited data to guide treatment approaches. It demonstrates successful reversal of amenorrhea through tailored hormone therapy and adds to the evidence base for managing reproductive manifestations in this challenging syndrome.

## 4. Conclusions

To the best of our knowledge, this is the first case that explored the successful management of primary amenorrhea in a case of WSS over the course of four years. We presented this case to demonstrate the positive outcomes that could result from continuous treatment with hormonal replacement therapy. This case demonstrates the successful management of primary amenorrhea in a patient with confirmed Woodhouse–Sakati syndrome using incremental estrogen and progesterone therapy. Detailed evaluation and genetic testing led to the diagnosis of WSS with associated hypogonadotropic hypogonadism causing amenorrhea. Individualized hormone replacement therapy facilitated pubertal development and reversal of amenorrhea. This highlights the importance of prompt diagnosis and tailored treatment. Genetic counselling is also key. A multidisciplinary approach is ideal when managing rare disorders like WSS. Further studies are needed to develop optimal treatment guidelines.

The limitations of this study include that it is a single case report with a short follow-up duration. The treatment approach was empirical rather than evidence-based due to the lack of management guidelines for this rare syndrome. The results cannot be generalized to all patients with WSS and amenorrhea, as significant variability exists in clinical features and severity. Compliance and regular follow-up are important to ensure optimal outcomes. Long-term studies on larger number of patients are needed to develop standardized protocols for diagnosis and management. The psychological impact and quality of life implications also need further evaluation.

## Figures and Tables

**Table 1:** Comparison of our case’s clinical features with clinically diagnosed cases reported in the literature.

Clinical Features	Current Report	Alasiri et al. [17]	Louro et al. [18]	Alharbi, [19]
Alopecia	+	+	+	+
Primary amenorrhea	+	+	+	+
Absence of secondary sexual characteristics	+	+	+	+
Diabetes mellites	-	+	+	+
Sensorineural hearing loss	-	+	+	?
Extrapyramidal symptoms	-	?	+	+
Intellectual disability	+	+	+	+
Hypothyroidism	-	+	+	+
Triangular face	+	?	+	+
Pituitary hypoplasia	-	?	?	+
Breast hypoplasia	+	+	?	+
Hypergonadotropic hypogonadism	+	+	+	+
White matter disease	-	?	+	+
Urogenital anomalies	-	-	-	+
Bilateral keratoconus	-	-	?	+

**Table 2:** Summary of the patient’s laboratory findings at the ages of 15 and 19 years.

	At the Age of 15 Years	At the Age of 19 Years	Reference Value
Hemoglobin A1C	6.8	-	4.4–6.4%
Insulin level	72.9	-	3.2–16.3 uIU/mL
Thyroid Profile			
Free T4	13.50	9.90	7.50–21.10 pmol/L
Thyroid Stimulating Hormone (TSH)	3.266	1.87	0.38–5.33 uIU/mL
Hormonal Profile			
Follicle Stimulating Hormone (FSH)	48.5	17.69	Mid-Follicular Phase 3.85–8.78 mIU/mL Mid-Cycle Peak 4.54–22.51 mIU/mL Mid-Luteal Phase 1.79–5.12 mIU/mL
Luteinizing Hormone (LH)	17.96	10.3	Mid-Follicular Phase 2.12–10.89 mIU/mL Mid-Cycle Peak 19.18–103.03 mIU/mL Mid-Luteal Phase 1.20–12.86 mIU/mL
Prolactin	-	139	71.06–568.5 uIU/ml
Testosterone Total	-	1.07	0.35–2.60 nmol/L

## Data Availability

Data available upon request.
